# Astrocytes play a key role in activation of microglia by persistent Borna disease virus infection

**DOI:** 10.1186/1742-2094-5-50

**Published:** 2008-11-11

**Authors:** Mikhail V Ovanesov, Yavuz Ayhan, Candie Wolbert, Krisztina Moldovan, Christian Sauder, Mikhail V Pletnikov

**Affiliations:** 1 Department of Psychiatry and Behavioral Sciences, Johns Hopkins University School of Medicine, Baltimore, MD, USA; 2 Center for Biologics Evaluation and Research, the US Food and Drug Administration, Bethesda, MD, USA

## Abstract

Neonatal Borna disease virus (BDV) infection of the rat brain is associated with microglial activation and damage to certain neuronal populations. Since persistent BDV infection of neurons is nonlytic *in vitro*, activated microglia have been suggested to be responsible for neuronal cell death *in vivo*. However, the mechanisms of activation of microglia in neonatally BDV-infected rat brains remain unclear. Our previous studies have shown that activation of microglia by BDV in culture requires the presence of astrocytes as neither the virus nor BDV-infected neurons alone activate microglia. Here, we evaluated the mechanisms whereby astrocytes can contribute to activation of microglia in neuron-glia-microglia mixed cultures. We found that persistent infection of neuronal cells leads to activation of uninfected astrocytes as measured by elevated expression of RANTES. Activation of astrocytes then produces activation of microglia as evidenced by increased formation of round-shaped, MHCI-, MHCII- and IL-6-positive microglia cells. Our analysis of possible molecular mechanisms of activation of astrocytes and/or microglia in culture indicates that the mediators of activation may be soluble heat-resistant, low molecular weight factors. The findings indicate that astrocytes may mediate activation of microglia by BDV-infected neurons. The data are consistent with the hypothesis that microglia activation in the absence of neuronal damage may represent initial steps in the gradual neurodegeneration observed in brains of neonatally BDV-infected rats.

## Background

Borna disease virus (BDV) is a non-segmented, negative-strand RNA virus that persistently infects the central nervous system (CNS) and causes behavioral abnormalities in a broad spectrum of warm-blooded animals [1-3]. Intracranial inoculation of newborn rats with BDV leads to a persistent infection of neurons and astrocytes with minimal signs of classical inflammatory cell infiltration (e.g., encephalitis and meningitis), but is associated with a progressive loss of granule cells in the dentate gyrus of the hippocampus, Purkinje cells in the cerebellum, and GABA-ergic neurons in the neocortex [4-7].

BDV replicates slowly without inducing lysis of host cells[1,3,8]. The mechanisms of selective neuronal loss in neonatally BDV-infected rats remain unclear. Based on a temporal and regional association between neuronal damage and microgliosis, previous studies have suggested that activated microglia could contribute to BDV-associated neuropathology [9-11]. As BDV does not infect microglia *in vivo *or *in vitro*[11,12], and since BDV does not directly activate cultured purified microglia *in vitro*[12], dying BDV-infected neurons have been proposed to trigger microgliosis as a secondary response [13].

However, our previous *in vitro *study has demonstrated that persistent BDV infection of cortical cultures leads to activation of microglia in the absence of neural pathology, suggesting that activation of microglia precedes cell death [12]. Furthermore, we also found that astrocytes appear to be indispensible for the activation of microglia by BDV-infected neurons [12]. The present study sought to evaluate the mechanisms whereby astrocytes may contribute to BDV-mediated microglia activation. Using the mixed culture system, we show that non-cytopathic infection of neurons stimulates astrocytes that in turn are able to activate microglia. The present findings indicate that astrocytes play a key role in mediating activation of microglia by BDV infection in the absence of overt neuronal toxicity or direct infection of microglia.

## Methods

### Reagents

Lipopolysaccharide (LPS) from Escherichia coli 026:B6, staurosporine, Hoechst 33258, DNase, poly-L-lysine, laminin, rat interferon-α (IFN-α) and fluorescein isothiocyanate (FITC)-labeled isolectin I-B4 from Griffonia simplicifolia seeds (lectin IB_4_) were obtained from Sigma Chemical Co. (St. Louis, MO). Recombinant rat IFN-α was re-suspended in PBS and frozen in aliquots of 2.6 × 10^5 ^units/ml. A diluted stock solution was prepared in PBS (2.6 × 10^3 ^units/ml). Mouse anti-rat CD11b/c (clone OX42) monoclonal antibody was purchased from BD Biosciences (San Diego, CA). Rabbit anti-ionized calcium binding adapter molecule 1 (Iba1) antibody was obtained from Wako Chemicals USA (Richmond, VA). Goat polylonal anti IL-6 antibody was from Santa Cruz Biotechnology (Santa Cruz, CA). Chicken anti-microtubule associated protein 2 (MAP2) polyclonal antibody, rabbit anti-glial fibrillary acidic protein (GFAP), anti-ED1 MAB and the secondary antibodies carbocyanin (Cy) 3, Cy 5 or fluorescein isothiocyanate (FITC)-conjugated donkey anti-mouse, anti-rabbit and anti-chicken IgG antibodies were obtained from Chemicon (Temecula, CA). Monoclonal antibody directed against BDV protein N (Bo18) was a generous gift by Dr Juergen Richt, National Animal Disease Center, 2300 Dayton Avenue, Ames, IA [14]. Dulbecco's modified Eagle medium (DMEM) with high glucose (4,500 mg/l), DMEM/F12 (1:1) nutritional supplemented media, Neurobasal-A medium, serum-free B-27 supplement (NBM), heat-inactivated horse serum (HS), HEPES buffer solution (HBS), Hank's balanced salt solution (HBSS), L-glutamine solution, penicillin-streptomycin solution (P/S, 50 U/50 μg per ml), trypsin (0.25%)-EDTA (1 mM) and trypan blue were purchased from Invitrogen/GIBCO-BRL (Carlsbad, CA). Certified heat-inactivated fetal bovine serum (FBS) was obtained from Hyclone (Logan, UT). LPS stocks of 1 mg/ml were prepared in DMEM.

### Virus stock preparation and titration

Virus stock was prepared from human oligodendroglia cells (kindly provided by Dr G. Pauli, Institut für Virologie, Freie Universität Berlin, Germany) persistently infected with BDV strain He/80 as described previously [12]. Briefly, confluent 175-cm^2 ^culture flasks were washed with 20 mM HEPES (pH 7.4) and incubated with 20 ml of 20 mM HEPES containing 250 mM MgCl_2 _and 1% FBS for 1.5 hours at 37°C. Subsequently, supernatants were harvested and centrifuged twice at 2500 × g for 15 minutes to remove cell debris. Virus particles were concentrated by ultra-centrifugation for 1 hour at 20°C at 80,000 × g onto a 20% sucrose cushion containing 20 mM HEPES and 1% FBS. Virus-containing pellets were resuspended in PBS to approximately 10^7 ^focus-forming units/ml. Determination of viral titers was carried out on C6 rat glioma cells (ATCC, Manassas, VA) by an immunofocus assay with the monoclonal anti-BDV antibody Bo18 as described previously [12]. Mock stock was prepared from uninfected Oligo cells as described above for BDV stock.

### Cell culture

#### Pure microglia and astroglia cultures

All experiments were performed with adherence to the National Institutes of Health guidelines on the use of experimental animals and the protocols approved by the Johns Hopkins Medical Institutions' Research and Animal Care Committee. To prepare mixed glial cultures, cerebral cortices from 1–3-day-old Fisher344 rats (Harlan, Indianapolis, IN) were surgically removed and placed in cold DMEM, the meninges carefully separated, and cortices minced and dissociated with trypsin-EDTA at 37°C for 15 minutes. After tituration with DNase (3 μg/ml), the mixed glial cell suspension from 2 brains was plated in a poly-L-lysine coated 75-cm^2 ^vented cell culture flask with DMEM/F12 medium supplemented with 10% FBS and 1% (v/v) P/S and grown in a humidified 5% CO2 incubator at 37°C. On days 14 to 21 in vitro, microglia were detached using an orbital shaker (150 rpm, 7 h, 37°C), centrifuged (150 × g, 15 minutes), and microglia number and viability were assessed by trypan blue exclusion. Depending on the particular experimental protocol (see below), microglia were plated in either 96-well cell culture plates, 24-well cell culture plates, or cell culture inserts in DMEM supplemented with 10% FBS and 1% (v/v) P/S, and placed in a humidified 5% CO_2 _incubator at 37°C.

To prepare pure astroglia, mixed glial cultures were washed every other day with fresh medium to remove loosely attached microglial cells and inhibit microglia growth. After 2–3 weeks in culture, glial cultures were passaged (1:4 split ratio) using trypsin (0.25%), and allowed to form a confluent monolayer for 3 to 7 days. This monolayer consisted of ~90–97% astrocytes and was used for infection with BDV. Infected cells were fed every week and passaged no more then 3 times or 2 months post infection.

#### Cortical neuron-rich cultures

Neuron-rich cultures were prepared from cortices of Fisher344 rats (Harlan, Indianapolis, IN) at embryonic days 19–20 using standard techniques [12,15]. Briefly, meninges-free cortices were isolated, trypsinized and mechanically dissociated by passing through fire-polished Pasteur pipettes. Washed cells were plated onto poly-L-lysine (0.05 mg/ml) and laminin (0.1 mg/ml)-coated tissue culture coverslips (200,000 cells/cm^2^) in Neurobasal-A medium with L-glutamine, B27 and P/S supplements (complete neurobasal medium, NBM). On day 2 in vitro, cells were infected with BDV or mock stocks diluted with fresh media with a multiplicity of infection (m.o.i.) of 0.02. Medium was partially (50%) replaced every 4th day thereafter. The composition of major cell types in the cultures was estimated by visual counting of cells immunostained with anti-MAP2 (neuron) or anti-GFAP (astrocytes) antibodies. Neuron-rich cultures contained > 95% neurons and < 4% glia.

#### Cortical mixed neuron/glia cultures

For preparation of mixed neuron and glia cultures, cortices from E15–E16 embryos (Fisher344 rats) were used as described previously [12,16]. Dissociated cells (350,000 cells/cm^2^) were cultured in DMEM/F12 supplemented with 5% FBS, 5% HS and 1% P/S (v/v) and otherwise treated as described above for neuron-rich cultures. Typical mixed neuron-glia cultures contained more astrocytes than neurons, astrocyte:neuron counts were split 2:3.

### Neuronal cell lines

Rat neuronal cell line, derived from mesencephalus (AF5, Sprague-Dawley rat origin [17,18]) was generously provided by Dr. W.J. Freed (National Institute on Drug Abuse, Baltimore, MD); medullary thyroid carcinoma CA-cell line (CA77) [19] was generously provided by Dr. A.F. Russo (University of Iowa, Iowa City, IA). All cells were cultured in DMEM/F12 medium supplemented with 10% FBS and passaged every 3–10 days, upon reaching confluence. Cells were infected at m.o.i. of 0.05. Persistently BDV- or mock-infected cells were evaluated as sister cultures of the same passage, passaged in parallel, and used for experiments at passages 5–10 *p.i*.

### Preparation of conditioned medium

All experiments were performed on sister cultures infected with either mock or BDV. Conditioned media from mock/BDV cells were purified from cell derived particles by centrifugation (5,000 g, 20 min, +4°C) and filtration through a 0.22 μm filter and stored at +4°C for up to 48 h. Half of the medium was incubated at 65°C in a water bath for 2 h to inactivate the virus. For some experiments, the medium was boiled in a microwave (2 kW, 30 s). For co-culture experiments, conditioned medium was mixed 1:1 with fresh media. Successful complete heat inactivation of the virus was confirmed by an immunofocus virus titration assay.

### Cytokine/chemokine ELISA

Assays were performed on stored frozen (-70°C) supernatant samples obtained at time points indicated in the text. Cytokines IL1-β, IL6, IL10, TNF-α, and RANTES were determined using a sandwich ELISA (R&D Systems, Minneapolis, MN). Twenty five μl of media from a microglia culture were incubated with 50 μl of assay diluent in the assay plate for 2 h at room temperature. After washing away any unbound substances, horseradish peroxidase-linked polyclonal anti-cytokine antibody was added to the wells. Following the addition of the peroxidase substrate solution, the enzyme reactive color product was detected by a microplate reader set to 450 nm with wavelength correction set to 540 nm.

### Multiplex cytokine assay

The rat 23 cytokines/chemokines LINCOplex (IL-1a, IL-1β, IL-2, IL-4, IL-5, IL-6 IL-10, IL-18, GM-CSF, GRO/KC, IFN-gamma, MCP-1, TNF-α, IL-9, IL-13, IL-17, Eotaxin, G-CSF, Leptin, MIP-1α, IP-10, RANTES, VEGF) from Millipore was used. The LINCOplex assay was conducted as per the manufacturer's instructions (Linco Research, Inc., St. Charles, MO). In brief, the assay is based on conventional sandwich assay technology. The antibody specific to each cytokine is covalently coupled to Luminex microspheres, with each antibody coupled to a different microsphere uniquely labeled with a fluorescent dye. The microspheres are incubated with standards, controls, and samples (25 μl) in a 96-well microtiter filter plate for 14 h at 4°C. After incubation, the plate is washed to remove excess reagents, and detection antibody, in the form of a mixture containing each of the 24 antibodies, is added. After 2-hr incubation at room temperature, streptavidin-phycoerythrin is added for an additional 30 min. After a final wash step, the beads are resuspended in buffer and read on the Luminex 100 instrument to determine the concentration of the cytokines of interest.

### ATP quantification

Adenosine 5'-triphosphate (ATP) determinations were performed using a bioluminescent luciferase ATP assay kit (Sigma Aldrich) according to manufactures recommendations as previously described [20]. Media samples were collected from confluent mock- or persistent or acutely infected cultures grown in 6-well dishes for 7 days. Cell homogenates were prepared from the PBS-washed cultures by scrapping the cells off the bottom of the dishes with a cell scraper and spinning at 4°C degrees at 3000 g for 5 minutes to remove excess of PBS. Cell culture media and pellet samples were stored at -80°C in 200 μl aliquots.

### IFN-α biological activity assay

Activity of type I interferon (IFN-α) in conditioned culture media was evaluated by its ability to inhibit replication of vesicular stomatitis virus (VSV) in C6 cells. A recombinant VSV expressing the green fluorescent protein (GFP) [21] was a generous gift from John Hiscott, PhD, McGill University, Montreal, Canada, Dr Peter Collins and Dr Ulla Buchholz, NIH, Bethesda, MD, USA. C6 cells were maintained in DMEM +9% FBS; 1.25 × 10^5 ^cells were seeded into 24 wells. Sixteen hours later, cells were pre-treated with samples of mock or BDV-conditioned media collected as described for the ATP assay and diluted 4-fold in DMEM (300 μl of media per well). Controls wells were pre-treated with 0, 10, 100 or 1000 units of rat IFN-α. 24 hours later, C6 cells were challenged each with 2000 PFU of VSV-GFP (stock 2.5 × 10^8 ^PFU/ml; multiplicity of infection 0.005). Nine hours later, the number of cells expressing GFP was evaluated by fluorescence microscopy. To this end, 22–30 visual fields were evaluated using ×10 objective to view the entire outer area of a well of a 24-well plate under a fluorescent microscope (Nikon Eclipse TE2000-U). Individual GFP-positive cells and numbers of foci (defined as a group of 4 and more GFP-positive cells) were scored.

### Immunocytochemistry

Co-cultures were washed two times with PBS and then fixed for 25 minutes with either phosphate buffered saline (PBS) plus 4% (w/v) paraformaldehyde at room temperature. After permeabilisation with 0.1% Triton X-100 (Sigma), cultures were blocked for 2 h in PBS with 2% donkey serum (Chemicon) and further incubated with primary antibody mixtures overnight at 4°C in blocking solution. After washing extensively, the cultures were incubated with secondary antibodies in blocking solution for 1 hour at room temperature. Antibodies against the following proteins were used: MAP2 (1:1000 dilution) as neuronal marker; GFAP (1:800) as astrocyte marker; ED1 (1:100), CD11b/c (1:50) and Iba1 (1:800) as microglia markers; activated caspase 3 (1:200) as apoptotic marker; cytokine IL-6 (1:500) and MHC I (1:100), MHC II (1:100) as pro-inflammatory markers; and anti-BDV N (1:50). Immunostaining without primary antibodies or staining of uninfected cultures were used as negative controls. Microglia were also stained with lectin IB4 as described previously [12]. Following mounting with Gel/Mount medium (Electron Microscopy Sciences, Hatfield, PA), the samples were allowed to dry in the dark and were viewed using a 40× objective lens on a LSM-5 confocal microscope from Carl Zeiss (Melville, NY) or Nikon Eclipse E400 equipped with the digital camera CoolSnap™ ES (Roper Scientific Production). Digital images of the cells were captured and processed by using standard software supplied with the microscopes. All sister culture samples were stained in parallel and imaged under the same parameters (exposure time, digital gain, magnification) to allow quantitative comparison of digital images. Digital intensity of staining was analyzed in at least 10 microscopic fields per sample.

### Semi-automated counting of activated microglia

Activated microglia were stained with lectin-FITC and imaged on a Nikon-Eclipse microscope to determine activated microglia numbers. The bright lectin-labeled round-shaped microglia had remarkably consistent size and shape appearance, and represented a subpopulation of activated, strongly ED1 and CD11b-positive microglia cells (see text). Lectin weakly stained resting ramified 'resting' microglia, but these cells were barely distinguished from a weakly lectin-positive background that was likely due to stained astrocytes. Therefore, round-shaped lectin-positive cells can be easily counted automatically. Round microglia cells are considered to represent the end-stage of microglia activation [12]. In addition, uniformly-sized round cells are easy to count by an unbiased observer. This approach helps exclude multiple intermediate species of microglia that seem to be at various stages of activation. Digital images were coded and counted semi-automatically using the "threshold" and "count particles" tools of the ImageJ software (NIH, Bethesda, MD). The threshold was set to label only round-shaped microglia cells, and the particles detection parameters were chosen to minimize counting of artifacts (i.e. bright objects less or significantly larger than a typical microglial cell). At least 10 random fields were evaluated for each culture.

### Statistical analysis

All experimental results are presented as mean ± SEM of BDV/mock ratios, with each value being obtained from a pair of sister cultures treated with media collected from BDV- or mock-infected cells, or BDV and mock-treated virus preparations. Experiments were repeated at least three times. Each experiment included two-five independent sister cultures, with each culture being plated in at least two-five wells per treatment condition. The data was analyzed with paired t-test for comparisons between two related groups (mock and BDV sister cultures). P value below 0.05 was used as the indication of significant differences.

## Results

### Round-shaped microglia formation represents microglia activation in BDV-infected brain cultures

**Figure 1:**
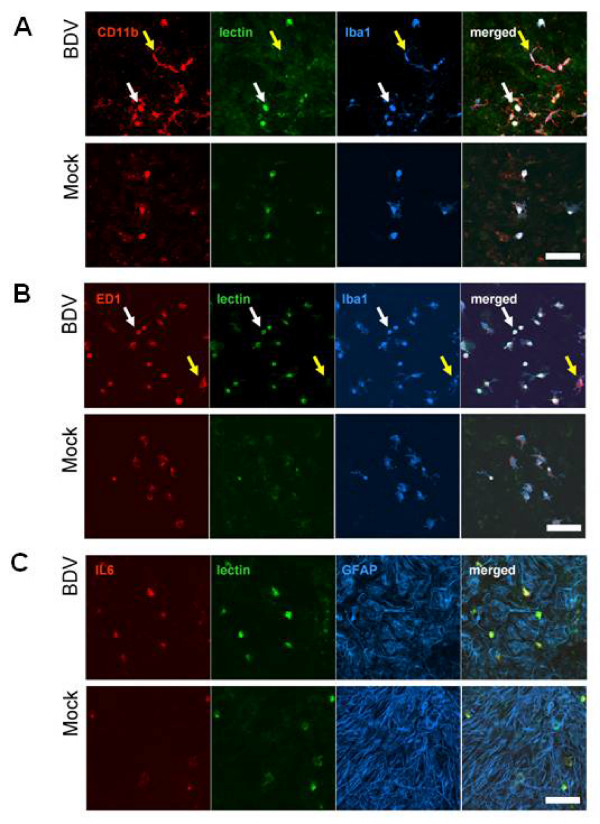
**BDV infection induces formation of round 'activated' microglia in mixed cultures**. Mixed neuron-glia-microglia cultures were BDV- or mock-infected on the 1st day in vitro (DIV), fixed on the 10th DIV and labeled with microglia-specific markers Iba1, CD11b, ED1 and lectin, or with astrocyte-specific marker GFAP, or a pro-inflammatory cytokine, IL-6. A – Co-localization of CD11b, lectin and Iba1 in round microglia (white arrows) but not in resting CD11b+ microglia (yellow arrows). B – Co-localization of ED1, lectin, and Iba1 in round microglia (white arrows) but not in resting ED1+ microglia (yellow arrows). C – Positive staining for IL-6 in round microglia (white arrows) but not astrocytes. Scale bar – 100 μm. The quantitative analysis of round microglia counts is shown in Figure 2A (the first column).

To investigate how BDV infection of neurons could activate microglia, we used neuron-glia-microglia cultures, in which BDV infection is manifested by high numbers of 'round' microglia cells that are morphologically different from resting 'ramified' microglia [12]. To find an optimal way of quantifying the microglia response to infection, we compared four markers of rat microglia (Figure 1a and 1B). In infected mixed cultures, IB_4_-lectin labeled only round microglia, whereas Iba1, CD11b, and to a lesser extent, monocyte-specific ED1 staining was present in both 'round' and 'ramified' microglia, indicating that 'round' microglia and 'ramified' microglia are two subpopulations of the same cell type (Figure 1A and 1B). Individual microglial cells in mock and BDV cultures did not differ in the distribution of the ED1, CD11b, Iba1 and IB_4 _staining, however BDV cultures contained significantly more round shaped microglia cells, consistent with our previous observations [12]. In addition, strong labeling for MHC-I and II (data not shown), and a pro-inflammatory cytokine, IL-6 (Figure 1C) indicated that round-shaped lectin-positive microglia were functionally activated. Thus, BDV-induced activation of microglia in mixed cultures can be reliably evaluated by assessing numbers of lectin-positive round-shaped cells.

### Microglia are activated by BDV-infected astrocytes

Our previous studies have demonstrated that the presence of astrocytes in mixed cultures appeared to be an important factor for BDV-associated activation of microglia [12]. Specifically, in contrast to mixed neuron/astroglia/microglia cultures, co-culture of pure BDV-infected neurons and pure microglia does not produce microglia activation. This data is in line with prior studies that have shown that astrocytes play a role in activating microglia during different neuroinflammatory and neurodegenerative processes [22,23].

**Figure 2:**
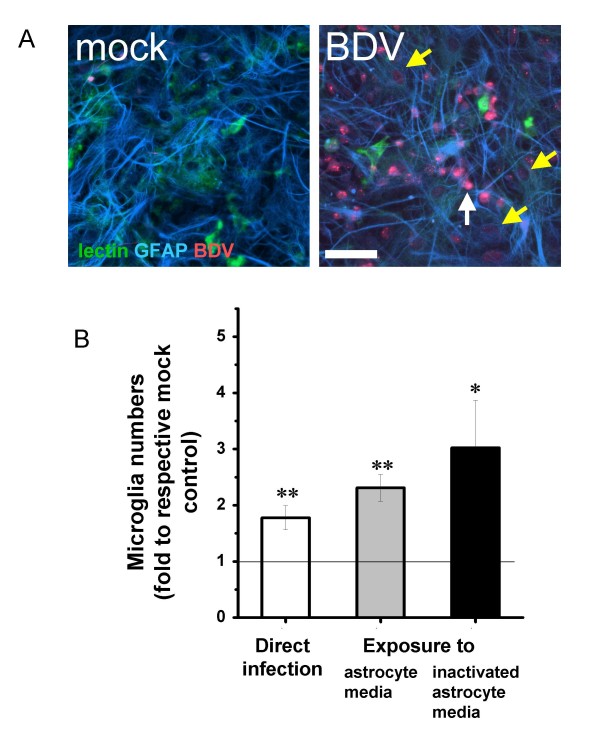
**Microglia are activated by conditioned media from BDV-infected astrocytes**. A – Mixed cultures were mock-or BDV-infected and stained with GFAP and BDV antibodies and lectin at 14 days p.i. Note that the astrocyte monolayer is a dominating cell surface in culture, providing bedding for microglia and neurons. Infected neurons can be identified as bright red round cells (white arrow). Many astrocytes are infected (yellow arrows). Note the absence of BDV infection of microglia (green stain). Scale bar – 50 μm. B – The numbers of round-shape activated microglia in 14 DIV mixed cultures infected on the 2nd DIV (white column); exposed to 2-day old conditioned media collected from BDV-infected astrocytes on the 10th DIV (gray column), or exposed to heat-inactivated media collected from BDV-infected astrocytes (black column). Each bar represents mean ± SEM (n = 5–12 experiments). The data are presented as the number of round microglia cells in BDV versus mock-treated sister mixed cultures. The results of the paired t-test on two populations (mock vs. mean) are shown as asterisks (*p < 0.05; **p < 0.005) Scale bar – 50 μm

Unlike microglia, astrocytes are susceptible to BDV infection and both neurons and astrocytes constitute BDV hosts in mixed neuron/microglia/glia cultures (Figure 2A) [11,24]. Therefore, one mechanism whereby astrocytes might activate microglia in mixed cultures is through BDV infection of astrocytes themselves. To evaluate this possibility, we seeded mixed neuron/astroglia/microglia cells over mock- or BDV-infected astrocytes and found that direct contact with BDV-infected astrocytes produced 1.97 ± 0.63 (mean ± S.D.) times more round microglia cells as compared to mock-infected astrocytes (Figure 2B).

To evaluate if direct contact between microglia and infected astrocytes is required, we compared microglia activation in mixed cultures either infected with BDV or exposed to conditioned medium from mock- or BDV-infected astrocyte cultures (Figure 2B). Conditioned medium from BDV-infected astrocytes produced a strong microglia activation that was even greater compared to that found in mixed cultures directly infected with the virus (Figure 2B gray column versus white column). Furthermore, to rule out a contribution of BDV infection transferred from pure astrocytes to mixed cultures, conditioned media were pre-treated with heat (30 min, 65°C). Heat inactivation did not inhibit microglia activation (Figure 2B black column versus gray column). In a separate experiment, large cell-derived components precipitated by centrifugation of BDV-conditioned medium were found not to induce microglia activation (data not shown). Taken together, the presented data suggest that microglia might be activated by small soluble heat-resistant substance(s) released by BDV-infected astrocytes.

### Activation of astrocytes by BDV infection

**Figure 3:**
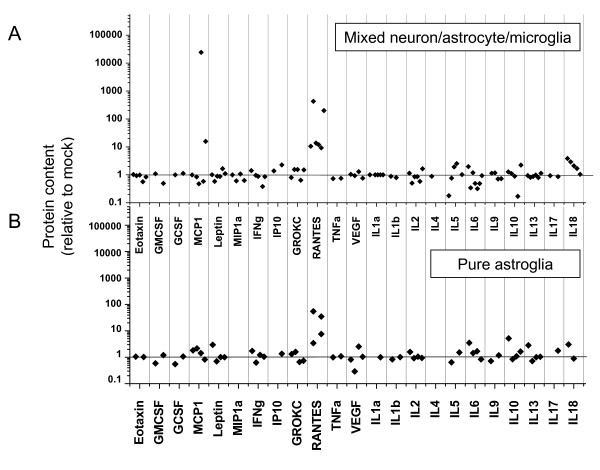
**The effects of BDV infection on expression of cytokines and chemokines in astrocyte or mixed cultures**. Supernatants were analysed for cytokine/chemokine protein contents using the rat 23 cytokines/chemokines LINCOplex assay. Levels of cytokines and chemokines in media samples from BDV-infected cultures normalized to the values in obtained from respective mock-infected sister cultures. Media samples were collected from n = 6 mixed astrocyte/neuron/microglia cultures at 12–14 d.p.i. and from n = 4 persistently infected astrocyte cultures at 7–10 days after passage.

Activated astrocytes have been demonstrated to actively participate in promoting neuroinflammation [e.g., [25]]. To evaluate activation of astrocytes by BDV infection, we assessed the profiles of cytokine/chemokine protein expression in BDV-infected mixed neuron/glia/microglia and BDV-infected purified astrocyte cultures using the multiplex assay, LINCOplex. Among 23 analytes, only RANTES was significantly up-regulated in both culture preparations, providing a molecular marker of BDV-induced activation of astrocytes (Figure 3).

**Figure 4:**
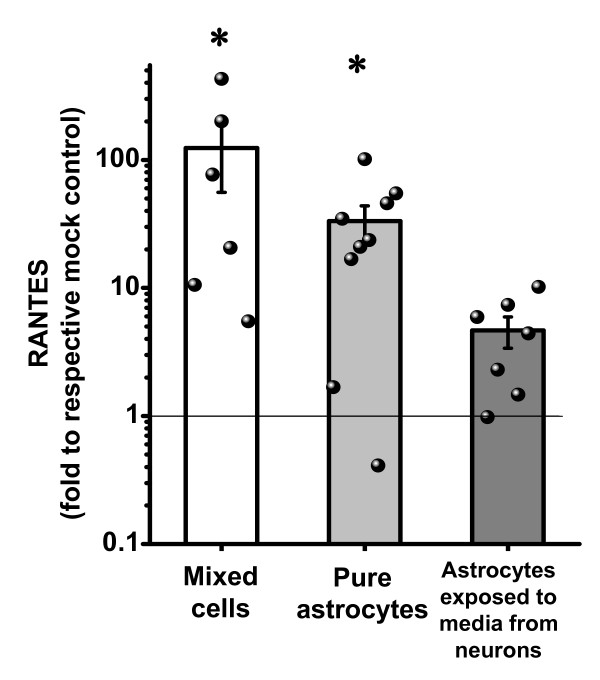
**Astrocytes are activated by BDV and conditioned media from BDV-infected neurons**. Measurement by ELISA and multiplex of the release of RANTES by BDV-infected mixed cultures (white bar), persistently BDV-infected pure astrocytes (gray bar) or after exposing primary uninfected astrocytes to media collected from BDV-infected neuronal cells (dark gray bar). Media were collected at 10–14 d.p.i. for mixed cultures, at 7–10 days after passage for persistently infected astrocytes, and at 2–4 days after AF5-media treatment for astrocyte cultures. Each bar shows mean ± SEM normalized to values obtained from respective mock-treated sister cultures. Dots indicate single data values for each experiment. The results of the paired t-test on two populations (mock vs. mean): *p < 0.05

However, in contrast to pronounced BDV infection of astrocytes in culture (Figure 2A), astrocytes *in vivo *largely avoid BDV infection during the first month after inoculation [9-11]. Since microgliosis and astrogliosis are evident in the brain one week after inoculation with BDV [26,27], direct BDV infection of astrocytes is unlikely a factor in mediating microglia activation *in vivo*. In order to evaluate whether BDV-infected neurons can activate astrocytes without infection of the latter, we exposed primary uninfected astrocytes to heat-inactivated 2-day conditioned medium from mock- or persistently BDV-infected AF5 neuronal cells. AF5 neuronal cells grown in the same media as astrocytes were chosen for this experiment because our pilot experiments had shown that serum-free medium from primary purified neurons was toxic to microglia and astrocytes. Treatment of astrocytes with media collected from BDV-infected neurons induced a 4.7 fold higher up-regulation of RANTES as compared to astrocytes treated with media from mock-infected neurons (Figure 4). This indirect activation of astrocytes, however, was lower than the 33-fold increase in RANTES expression (BDV vs. mock) by directly infected astrocytes and the 124-fold increase by infected mixed neurons/astrocyte/microglia cultures (Figure 4). Taken together, our findings demonstrate that heat-inactivated conditioned medium from BDV-infected neurons induced activation of uninfected astrocytes *in vitro*.

### Astrocytes mediate activation of microglia by infected neurons

**Figure 5:**
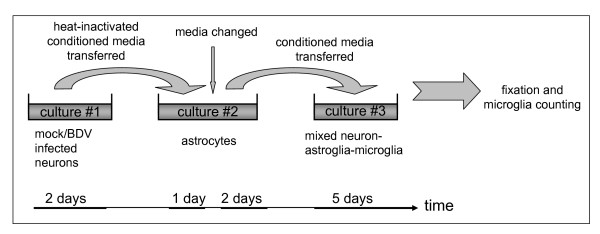
**Activated astrocytes produce soluble factors that activate microglia**. The experimental design: Two-day old conditioned media collected from sister mock- or BDV-persistently infected neuronal cultures (culture #1: AF5, CA77 or primary neurons) were heat-inactivated and transferred to primary astrocytes (culture #2) to induce astrocyte activation. 24 hours after transfer of media, activated astrocytes were washed, fresh medium was added and collected one day later. This astrocyte conditioned medium was transferred to mixed neuron/astrocyte/microglia (culture #3) to induce microglia activation.

**Figure 6:**
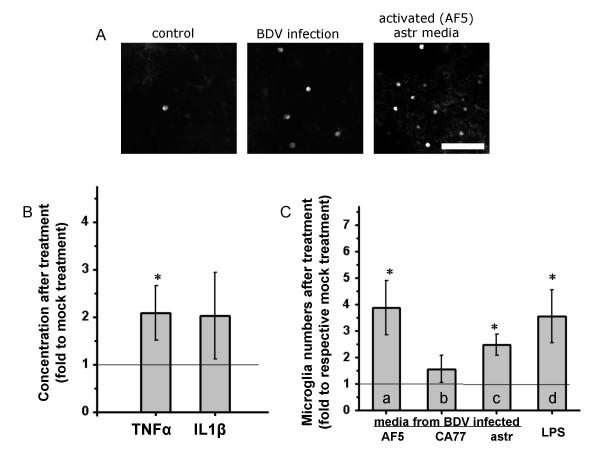
**Astrocytes activated by BDV-infected neurons induce microglia activation in mixed cultures**. A – Lectin staining of round microglia in mixed cultures left either uninfected and untreated (control), or BDV-infected and untreated, or uninfected but treated with conditioned media from AF5-activated astrocytes. Scale bar – 50 μm. The quantitative data for AF5-activated astrocytes are presented in C (column *a*). B – LPS-induced production of TNF-α and IL-1β by mixed cultures. TNF-α and IL-1β were measured by ELISA. Note a significant up-regulation of the cytokines in mixed cultures activated by conditioned media from astrocytes pre-treated with media collected from BDV-infected AF5 neuronal cells. Values are given as ratio compared to concentrations measured in mixed cultures exposed to conditioned media from astrocytes pretreated with media collected from mock infected AF5 neuronal cells, n = 3–5. C – Activation of microglia in mixed cultures following exposure to medium from astrocytes conditioned with medium from different types of infected neuronal cells. Astrocytes can be activated by persistently BDV-infected neuronal AF5 cells (a) or CA77 cells (b). Medium from persistently BDV-infected astrocytes (c) as well as LPS treatment of mixed cultures (d) were used as positive controls. Conditioned media from different pre-treated astrocyte cultures were added to mixed cultures to activate microglia. The results are numbers of round microglia cells in BDV-treatments normalized to respective mock-treated sister cultures, n = 3–6. The results of the paired t-test on two populations (mock vs. mean): *p < 0.05.

To investigate if astrocytes activated by BDV-infected neurons could in turn stimulate microglia activation, mixed cultures were treated with conditioned media from pure astrocytes that were pre-treated with conditioned media collected from mock- or BDV-infected neuronal AF5 cells (see Figure 5 for the experiment design). Compared to mixed cultures treated with media from astrocytes per-treated with media from mock-infected neurons, we found a 3.9-fold increase in the number of round microglia cells in mixed cultures treated with conditioned media collected from astrocytes pre-treated with media from BDV-infected neuronal cells (BDV-AF5-astrocyte-treated mixed cultures (Figure 6A). To confirm functional activation of round shaped microglia, we stimulated mixed cultures with LPS and observed a significantly larger release of TNF-α and IL1-β by BDV-AF5-astrocyte treated cultures vs. mock-AF5-astrocyte treated mixed cultures (Figure 6B). We also found that astrocyte activation was not limited to the AF5 neuronal cell line and was also observed after treatment of astrocytes with media collected from the BDV-infected CA77 neuronal cell line or from infected primary cortical neurons (Figure 6C). Furthermore, we used mock or BDV-infected primary cortical neurons at 10 d.p.i. in a similar experiment. Since media for primary astrocytes and primary neurons are incompatible, primary neurons were switched to serum-containing media one day before the experiment. Such conditioned media from primary BDV-infected neurons activated astrocytes as evidenced by their (i.e., astrocytes) ability to produce a 2.54 ± 0.86-fold increase in numbers of round microglia cells in mixed cultures compared to mixed cultures treated with media collected from astrocytes pre-treated with media from mock-infected primary neurons.

### Soluble factors that may mediate activation of astrocytes and microglia

Our present experiments clearly demonstrate that soluble factors released by BDV-infected neurons or activated astrocytes likely play a role in cell-to-cell communications in culture. Thus, we performed an initial characterization of potential mediators of neuron-astrocyte-microglia interactions.

### Activation of astrocytes by BDV proteins

Astrocytes can be activated by viral proteins and other pathogens (e.g., HIV-encoded Tat protein or LPS [28,29].) As conditioned medium from BDV-infected neurons is infectious and since this infectivity is abolished by heat-inactivation (data not shown), BDV proteins released by infected neurons could potentially contribute to activating astrocytes. To investigate if BDV proteins can activate astrocytes, we used increasing amounts of virus stock to infect mixed neuron/astroglia/cultures (data not shown). 4 days after infection, virus spread to the majority of neurons and the percentages of infected neurons were comparable between wells exposed to 1, 5 or 20 μL of the virus stock per 1 mL of media (data not shown). In contrast, the number of round-shaped microglia cells proportionally rose with the increase in the concentration of the stock used, suggesting that despite the equivalent infectivity of neurons, more virus proteins delivered with virus stock induced more activation of microglia. Furthermore, to evaluate if astrocytes can be directly activated by BDV proteins without being productively infected by BDV, BDV or mock stocks were exposed to UV light for 40 min and the inactivated mock/BDV stocks were applied to astroglia cultures (no astrocyte infection was observed, data not shown). Conditioned media collected from treated astrocytes were able to induce microglia activation in mixed neuron/astroglia/microglia cultures (6.3 ± 0.9 vs. 11.8 ± 2.6 round microglia per field, mock vs. BDV treatment). Moreover, treatment of mixed cultures with LPS led to secretion of a greater amount of TNF-α upon exposure to media collected from astrocytes treated with inactivated BDV particles compared to mock stocks (data not shown). Thus, our results indicate that BDV proteins could mediate activation of astrocytes in culture.

### Soluble factors that activate astocytes or microglia

Several soluble factors released by astrocytes can induce microglia activation in culture, including cytokines and chemokines (e.g., RANTES, M-CSF, IL-6) [21]. We found no significant alterations in levels of 23 soluble factors in culture media from astrocytes pre-treated with media from BDV-infected AF5 cells using a 23 cytokine/chemokine multiplex assay (data not shown). Finally, heat-inactivation of culture media collected from BDV-infected astrocytes had no effect on the ability of the media to activate microglia in mixed cultures (Figure 2B, black column versus gray column). This result suggests that putative soluble factors that mediate activation of microglia are heat-resistant soluble factors. Despite that fact that this heat-resistance requirement seems to exclude the majority of cytokines or chemokines, inteferons of type I could still remain functional after heat treatment [30]. Although recent studies have demonstrated that genomic RNAs from some negative-strand RNA viruses, including BDV, do not trigger interferon induction [31,32], we evaluated the effects of media collected from AF5 (BDV or mock) cells on replication of VSV that is highly susceptible to the antiviral action of interferon (IFN). As expected, our positive control experiments demonstrated that pre-treatment of rat C6 astrocytes with rat IFN-α at the doses of 10, 100 or 1000 units/ml dose-dependently inhibited replication of VSV-GFP, as evidenced a dramatic decline in the number of GFP-positive cells. In contrast, media collected from mock- or BDV-infected AF5 cells had no significant effects on replication of VSV, leading to lysis of C6 cells, similar to VSV-infected C6 cells left untreated (data not shown).

Certain nucleotides, notably ATP, have been shown to activate astrocytes or microglia [33-36]. We used a bioluminescent luciferase assay to measure ATP concentration in media samples collected from BDV- or mock-infected neuronal cell lines. Neither acute nor persistent BDV infection of AF5 neuronal cells altered ATP concentrations in culture media: AF5 mock – 1.12 ± 1.61 nM vs. AF5 BDV – 1.27 ± 1.77 nM (n = 13); CA77 mock – 1.00 ± 0.87 nM vs. CA77 BDV – 1.09 ± 1.20 nM (n = 6). Similar data were obtained for media from primary astrocytes, primary mixed cells exposed to media from astrocytes, primary mixed cells exposed to media from primary astrocytes pre-treated with media from AF5 cells, or primary astrocytes exposed to media from AF5 neuronal cells (data not shown). Furthermore, we found that neither persistent nor acute BDV infection changed intracellular concentrations of ATP as measured in cell pellets from AF5, CA77 or PC12 cells (data not shown). As expected, intracellular concentrations of ATP in various neuronal cells were approximately 10–100-fold higher than concentrations of ATP in corresponding culture media.

## Discussion

The present study demonstrates that astrocytes play a key role in activating microglia by BDV infection. Our data suggest the following sequence of events that mediate activation of microglia in the course of BDV infection: BDV-infected neurons may release soluble heat-resistant molecules or virus-associated particles and/or viral proteins that can activate uninfected astrocytes as evidenced by expression of RANTES. Activated astrocytes in turn are able to stimulate microglia that acquire a round shape, express MHC I, MHC II and IL-6, and display increased secretion of TNF-α and IL1-β upon LPS priming. Activated astrocytes may secrete soluble heat-resistant low molecular weight factors that activate microglia.

Intracranial inoculation of BDV into newborn rats is associated with a gradual loss of a) granule cells of the dentate gyrus of the hippocampus, b) Purkinje cells in the cerebellum and c) GABA-ergic neurons in the cortex and striatum [1,7,13]. However, the mechanism of BDV-associated neuronal loss is poorly understood as, unlike neurotropic lytic virus infections (e.g., cytomegalovirus, Semliki forest virus), BDV does not induce any apparent toxicity to neurons that are the primary host cells in the CNS [8,12]. Given the overall strong temporal and regional association of neurodegeneration and microgliosis in BDV-infected rat brains, it is tempting to speculate that microglia activation results from BDV-induced cell death. Notably, reactive gliosis is a universal response of the innate immune system to brain pathology[37]. The exact functions of this phenomenon are not completely understood but the available data seem to indicate that reactive gliosis may support injured neurons and/or eliminate terminally damaged cells [38,39]. BDV infection, however, is non-lytic in culture, suggesting that activation of microglia by BDV infection is unlikely initiated by cell damage.

Our previous studies have demonstrated that neither exposure of microglia to the virus nor exposure of microglia to BDV-infected neurons leads to activation of microglia. It is the presence of astrocytes in mixed cultures that is required for microglia to be activated [12]. This result is consistent with prior reports that have demonstrated the importance of astrocytes for activation of microglia. For example, astrocytes have been found to enhance LPS-induced nitric oxide production by microglial cells [39], mediate microglia activation by trimethyltin [40], or modulate microglia signaling pathways during neuroinflammation [41]. Until recently, most studies interpreted the timing of activation of astrocytes to the effect that they rather play a secondary role in the initiation of events. However, there is a growing body of evidence that suggests an initiating role of astrocytes in neuroinflammation [38,42]. We believe that persistent BDV infection represents another example when astrocytes play a crucial role in triggering the innate immune response in the CNS and provides a valuable model for studying the molecular mechanisms of cell-to-cell communications in the context of chronic neuroinflammation and resultant neurobehavioral abnormalities.

Our findings further indicate that direct cell contacts between neurons and astrocytes are not required for activating microglia and that soluble factors secreted by neurons and astrocytes appear responsible for microglia activation. Since uninfected astrocytes need to be activated first to mediate ensuing activation of microglia, we explored what factors could be involved in this process. Factors produced by neurons in response to infection or injury include viral proteins and various chemokines and cytokines [43]. Based on the UV inactivation experiments as well as the enhanced astrocyte activation using increasing amount of virus stock, we speculate that certain viral BDV proteins may mediate activation of astrocytes. The data is in line with the observations that Tat or gp120 activate astrocytes by destabilizing intracellular Ca2^+^, subsequently leading to release of RANTES and IL-6 [28,44]. It should be pointed out that both mock and BDV virus stocks were prepared using the standard protocol [12] and contained virus particles as well as cellular and viral protein contaminants. Thus, activation of astrocytes could be due to virus related components such as particle-associated and free viral proteins. In contrast, a contribution of cellular material unrelated to BDV appears unlikely since mock and BDV stocks were purified and used under the identical conditions throughout the study. We found that treatment of primary astrocytes with media collected from BDV-infected neurons led to up-regulation of RANTES. These results are consistent with previous studies that demonstrated an up-regulation of IP-10 and RANTES in the brains of neonatally BDV-infected rats [45,46]. Interestingly, the authors have found that a likely source of chemokines in the infected brain parenchyma are astrocytes, compatible with our hypothesis that astrocytes are activated by BDV-infected neurons to initiate neuro-inflammatory cascades *in situ *as well.

Activated astrocytes have been shown to produce soluble factors that are able to activate microglia [22]. What activates microglia in the course of BDV infection remains obscure. In a search for possible candidates, we screened 22 soluble cytokines and chemokines using a mulitplex analysis. Only RANTES was found to be significantly up-regulated by BDV infection Furthermore, in our preliminary experiments, MG-CSF failed to induce round microglia formation under the conditions where BDV infection did (data not shown). Interestingly, heat inactivation of conditioned media from BDV-infected astrocytes (used as a positive control of astrocyte activation) had no effects on the ability of media to activate microglia, suggesting that putative soluble mediators are heat-resistant low-molecular weight factors. Possible candidates could include "gliotransmitters" such as ATP [47]. These molecules secreted by neurons and astrocytes work as mediators of cell-to-cell communication in a physiological condition, and may serve as "warning molecules" in pathological conditions [22,48]. We found no significant effects of BDV infection on production of ATP. In addition, the ATP concentrations in the media was very low (< 10 nM) compared to concentrations of 0.1–10 mM that have been found to activate neurons and microglia in vitro [33-36]. Noteworthy, ATP in culture media is typically associated with release of intracellular ATP from lysed cells. Thus, our findings are consistent with the lack of BDV-associated cell toxicity in vitro. Future studies may help to identify if other nucleotides mediate microglia activation.

Our data provide more insights into the possible sequence of events leading to neurodegeneration in neonatally BDV-infected rats. Primary BDV infection of neurons may produce activation of astrocytes and ensuing activation of microglia. It remains unresolved whether microglia activation initiates cell demise or simply exacerbates on-going cell injury. A recent report by Solbrig et al [49] lends an additional support for the first hypothesis. Specifically, the authors have shown that inhibition of microglia activation with a cannabinoid agonist decreases cell death in the BDV-infected rat brain [48]. In a broader context, our report is in line with an emerging idea that chronic activation of the innate immune system in the brain contributes to neuronal injury even if a pathogen may not be directly toxic to neurons.

## Conclusion

The present study demonstrates that astrocytes play a key role in activating microglia by BDV infection. We report that BDV-infected neurons activate uninfected astrocytes as evidenced by expression of RANTES, and activated astrocytes in turn stimulate microglia. Our study indicates that viral proteins could be responsible for activation of uninfected astrocytes that may secrete heat-resistant low molecular weight factors to activate microglia in mixed cultures.

## Abbreviations

BDV: Borna Disease Virus; VSV: vesicular stomatitis virus; IFN: interferon; d.p.i.: days post infection; PBS: phosphate buffered saline; ATP: adenosine 5'-triphosphate; GFP: green fluorescent protein; IL: Interleukin; LPS: lipopolysaccharide; FITC: fluorescein isothiocyanate; MAP2: microtubule associated protein 2; GFAP: glial fibrillary acidic protein; DMDM: Dulbecco's modified Eagle medium; HS: horse serum; HBS: HEPES buffer solution; P/S: penicillin-streptomycin solution; PBS: fetal bovine serum; HS: horse serum; SEM: standard error of mean.

## Competing interests

The authors declare that they have no competing interests.

## Authors' contributions

MVO, CS and MVP conceived and designed the study. MVO, AY, CW, CS and KM performed the experiments. MVO and MVP wrote the manuscript.
